# Oral delivery of protein and peptide drugs: from non-specific formulation approaches to intestinal cell targeting strategies

**DOI:** 10.7150/thno.61747

**Published:** 2022-01-01

**Authors:** Guanyu Chen, Weirong Kang, Wanqiong Li, Shaomeng Chen, Yanfeng Gao

**Affiliations:** 1 School of Pharmaceutical Sciences (Shenzhen), Sun Yat-sen University, Shenzhen, China.; 2 Department of Pharmacology and Pharmacy, Li Ka Shing Faculty of Medicine, The University of Hong Kong, Hong Kong SAR, China.

**Keywords:** Protein and peptide drug, Oral delivery system, Physical and biochemical barrier, Intestinal mucosa, Intestinal cell targeting, Oral bioavailability.

## Abstract

The past few years has witnessed a booming market of protein and peptide drugs, owing to their superior efficiency and biocompatibility. Parenteral route is the most commonly employed method for protein and peptide drugs administration. However, short plasma half-life protein and peptide drugs requires repetitive injections and results in poor patient compliance. Oral delivery is a promising alternative but hindered by harsh gastrointestinal environment and defensive intestinal epithelial barriers. Therefore, designing suitable oral delivery systems for peptide and protein drugs has been a persistent challenge. This review summarizes the main challenges for oral protein and peptide drugs delivery and highlights the advanced formulation strategies to improve their oral bioavailability. More importantly, major intestinal cell types and available targeting receptors are introduced along with the potential strategies to target these cell types. We also described the multifunctional biomaterials which can be used to prepare oral carrier systems as well as to modulate the mucosal immune response. Understanding the emerging delivery strategies and challenges for protein and peptide drugs will surely inspire the production of promising oral delivery systems that serves therapeutic needs in clinical settings.

## Introduction

**Figure 1:**
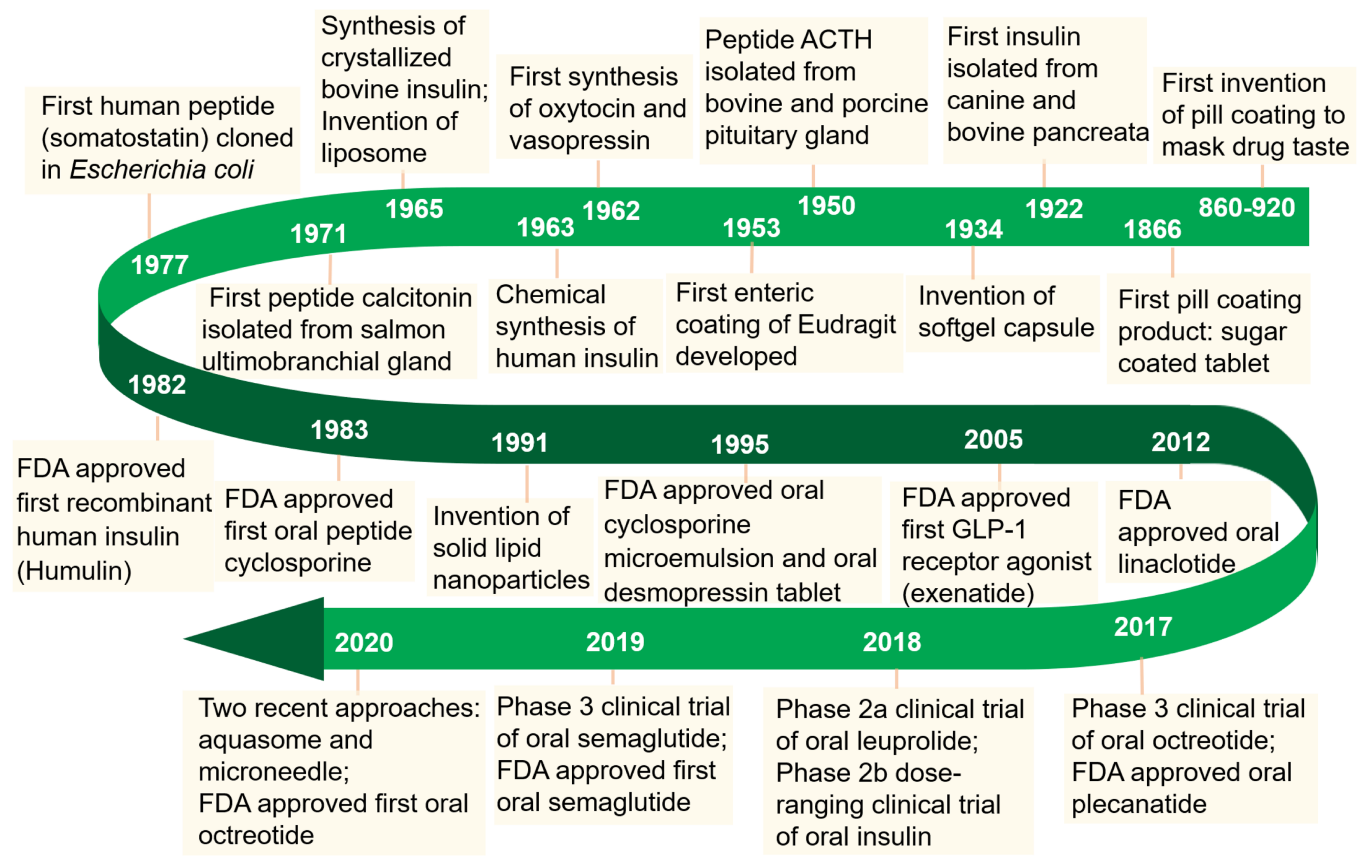
Milestones in the development of oral delivery of PPDs.

Enormous efforts have been made over the past few decades to realize the therapeutic efficacy of protein and peptide drugs (PPDs). Owing to their excellent specificity and biocompatibility, PPDs can achieve ideal therapeutic effects at relatively low doses 1. Since the isolation of insulin in 1922, the use of PPDs as therapeutic agents has been considered as an attractive approach to combat various diseases (**Figure 1**). Recent developments in the biotechnology and pharmaceutical sciences have made it possible to produce potential therapeutic PPDs in commercial quantities 2. By far, over 240 PPDs has been approved by FDA and a variety of potential drug candidates in clinical trials.

Though parenteral administration is the most commonly employed administration route for PPDs, it often associates with poor patient compliance 3. Compared to parental administration, oral drug delivery routes are advantageous in terms of patient compliance, safety, long-term dosing and manufacturing costs. Further, oral administration is used for both local and systemic delivery of a wide range of drug molecules, from small molecules to biomacromolecules 4. However, oral delivery of macromolecules (such as PPDs) is particularly challenging due to their physicochemical properties and the involving barriers in the gastrointestinal tract (GIT) 5. The major strategies to deliver PPDs orally with improved the therapeutic efficacy can be categorized into non-targeting and targeting delivery, including chemical modification and drug delivery systems for PPDs to avoid enzymatic degradation and reduce off-target drug distribution. Targeting different GIT area can be achieved by exploiting its physiological features and combining the PPDs with suitable drug formulations 6. Moreover, the presence of numerous types of intestinal cells, such as enterocytes, M cells, goblet cells and Paneth cells interspersed throughout the GIT provides various targets and allows for the design of a broad array of passive or active targeting delivery systems.

In this review, we summarize major barriers for oral delivery of PPDs, and the state-of-the-art formulation approaches for promoting the oral bioavailability of PPDs. Intestinal cell targeting strategies are presented with an emphasis on examples that showed great potential for clinical applications. Additionally, multifunctional biomaterials which can be used to prepare oral carrier systems as well as to modulate the mucosal immune response are also discussed.

## Physical and biochemical barriers and mechanism of intestinal drug absorption

The absorption of orally administered PPDs from the GIT into the systemic circulation is limited by various factors. These include the release of drugs from the carrier systems and pass on their way to the target receptors within the harsh intestinal environment. Ingested PPDs first encounter digestive enzymes in our oral cavity, including amylase and lipase in the saliva 7. The second enzymatic barrier is the intensive acidic environment and the presence of pepsin and cathepsin that degrades most of the PPDs in our stomach 8. Gastric pH might alter the ionization of the PPDs causing change of structure or function of the drug. Moreover, trypsin and α-chymotrypsin are the major proteolytic enzymes in the intestinal lumen 9.

**Figure 2:**
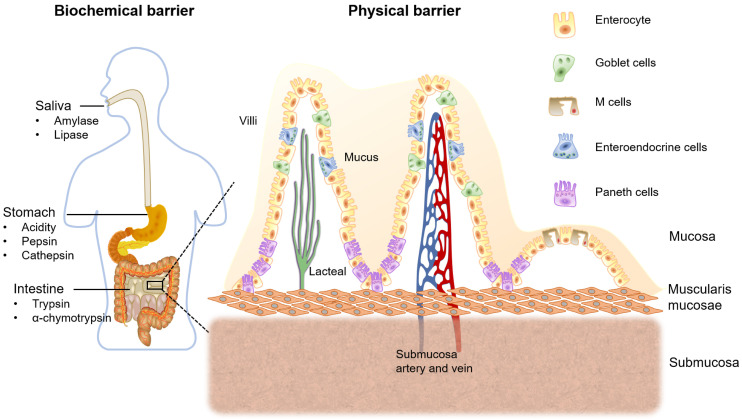
Biochemical and physical barriers for oral drug delivery, and the structure of intestinal mucosa with major intestinal cell types.

**Figure 2** shows the mucus layer covering GIT epithelial membrane is considered as the first physical barrier. Mucin is the main component which is a highly glucosylated glycoprotein. The backbone consists repeating sequences of serine, proline and threonine residues. The O-linked oligosaccharide side chains are generally terminated in L- fructose, sulfonic acid or sialic acid. Therefore, the intestinal mucus layer shows negatively charged 10, 11. Second physical barrier, the layer of epithelial cells connecting with tight junctions, which forming a seal wall for the drug permeation 12. Furthermore, PPDs being metabolized by the enterocytes cytochrome P450 3A4 (CYP3A4) enzyme and being pumped out via P-gp efflux protein, as well as the post-absorptive clearance are other involving barriers for oral drug delivery 13.

**Figure 3:**
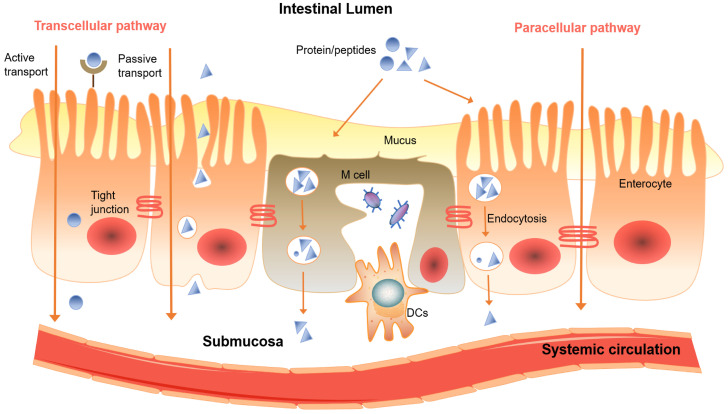
A diagram of transport pathways of protein and peptide compounds over the intestinal mucosal epithelial membrane.

The two major mechanism of drugs permeate through the intestinal mucosa are the passive diffusion via the transcellular or paracellular pathway (Figure 3), and the carrier-mediated transport including active transport and facilitated diffusion 14. The permeation mechanism for a particular drug depends on its physiochemical properties such as molar mass, polarity, lipophilicity and hydrophilicity 15, 16. Lipophilic, non-ionized form of drugs generally have higher permeability, while the ionized, hydrophilic drugs tend to penetrate over epithelium via paracellular pathway 17, and the hydrogen-bonding capability of the drugs dictated by the number of hydrogen bond donors and acceptors usually no more 10 and 5, respectively 18. Carrier-mediated transport is energy dependent, and has notable features of substrate specificity and saturability. It requires the interaction of drugs with a protein carrier often in the apical side of the intestinal membrane 19.

## Strategies to enhance oral bioavailability of PPDs

### Chemical modification

The oral bioavailability of PPDs is often hampered by their physicochemical characteristics, such as hydrophilicity, large molecular weight and sensitivity to enzymes and pH. To alter the physiochemical properties of PPDs, chemical modifications strategies, including lipidization, cationization, PEGylation and prodrug formation have been applied.

#### Lipidization

Rapidly and completely transported drugs are generally lipophilic and distribute readily into the epithelial cell membranes of GIT 20. The overall polarity of a drug molecule can be reduced by adding a non-polar or removal of a polar group to increase the lipophilicity, which leads to a higher concentration gradient for facilitating the diffusion of drugs over the intestinal mucosa. However, lipidization can reduce the water solubility of original drug. A typical drawback of lipidization is reduced receptor affinity 21.

One example is the leu-enkephalin peptide which is chemically modified by a reversible aqueous lipidization method with a dimethylmaleic anhydride analog. This resultant drug was stable in various pH phosphate buffers and showed greater stability against enzymatic degradation. The study demonstrated the lipidization may be an enabling strategy which can be used to enhance oral absorption 22. Nobex Corporation added a hydrophilic PEG chain (protection from enzymatic degradation) and a lipophilic alkyl chain to insulin for oral administration. Phase III results announced that it failed to meet the target endpoint, and recent iterations of PEG conjugation technique which include C_10_ and bile salts, presumably to promote peptide drug permeation. C_10_ elevates intestinal membrane fluidity via interaction with protein and lipids on the membrane, and it permeate over through both transcellular and paracellular pathways. However, Sakai *et al.* reported that high concentrations of C_10_ (>50 mM) could lead to significant cytotoxicity to Caco-2 cells, thus limiting the use of this technique 29. Additionally, it has been reported that lipidized drug inhibits the P-gp efflux pump. This strategy is particular suitable for Biopharmaceutical Classification System (BCS) class IV drugs that were reported to be easily effluxed by P-gp transporter 22.

#### Cationization

Cationic drugs are more permeable over the intestinal mucosa compared with anionic drugs, it is due to the negatively charged glycoproteins and glycosphingolipids on the intestinal cell membrane 23. Hence, formulating a cationic drug is postulated to elevate the drug permeability. However, peptide cationization may lead to increased immunogenicity, which will result in faster removal of the drug from the body and hence loss of activity. Moreover, its non-specific targeting in terms of tissue uptake, and potential toxicity found in the kidney and liver limits its therapeutic clinical use 23.

Studies have showed that PPDs can be cationized by chemical conjugation demonstrated efficient intracellular delivery via adsorptive-mediated endocytosis. **Futami** *et al.* demonstrated the negatively charged mammalian cell membrane consisting glycoproteins and glycosphingolipids, cationization of these proteins elevated their ability for intestinal drug permeation 24. Moreover, the recent developed sophisticated protein chemistry, controlled chemical modifications, such as substitutions, PEGylation and acylation, could significantly reduce side effects. Strategies to avoid protein misfolding and aggregation during storage are benefit in protein fibrillation. This in turn to prevent unforeseen side effects in drug delivery. 25. Thus, cationization has proven to be a great tool for oral PPDs delivery.

#### PEGylation

Generally, PEGylation is the covalent attachment of polyethylene glycol (PEG) to PPDs and elevate their half-lives due to steric hindrance against proteolytic enzymes. The increase in the molecular mass can improve both pharmacokinetic and pharmacodynamic properties of PPDs 26. However, PEG may lead to size enlargement, increased viscosity, or reduce cell affinity and limits the biological activity. Moreover, the non-biodegradable PEG materials might trigger adverse effects 27.

**Minimol** *et al.* have developed a PEGylated starch acetate nanoparticulate system for oral insulin delivery. An amphiphilic polymeric derivative was obtained by PEG conjugating with starch acetate, subsequently incubated with drug solution at the critical micelle concentration, forming self-aggregated drug loaded PEGylated nanoparticles. These self-aggregated nanoparticles showed only 32 nm in size allowing large surface area of the particles to contact with the intestinal mucosa. Moreover, the nanoparticles with great intestinal mucosal bioadhesion further promoted the drug permeation over the intestine 28.

#### Prodrug formation

A prodrug is a chemical derivative of a main drug, it usually has greater stability, solubility, lipophilicity and intestinal permeability. It converts to an active drug* in vivo* usually undergoes transformation either by a chemical or an enzymatic reaction. Esterification of hydroxyl, amino acid, or carboxylic acid containing drugs can increase lipophilicity, thus improve intestinal drug permeation 29, 30. However, the highly lipid-soluble drugs may bind to plasma protein, and limit free drugs in the plasma. Especially for PPDs, modification of PPDs maybe diminishes their specific receptors binding, since the plasma protein may occupy certain portion of the available PPDs. In some cases, during its activation stage, the prodrug might consume a vital cell constituent leading to its depletion.

#### Peptide cyclization and unnatural amino acids substitution

Cyclic cell-penetrating peptides (CPPs) show high stability and demonstrate great potential for the intracellular delivery 31. Cyclization normally improves the stability by removing exposed C and N termini of the peptides, which are susceptible to enzymatic cleavage. **Verdine** *et al.* 32 and **Clark** *et al.* 33 have proved that peptide cyclization by adding a lipophilic linker and enhanced oral absorption and drug stability. Our research team has previously developed a cyclic peptide C25 with disulfide bond by using a phage display technology targeting immune checkpoint LAG-3, and the cyclic peptide showed great stability and *in vivo* antitumor activity 34. Besides, our group previously had modified L-peptides to D-peptides 35, 36. As L-peptides are susceptible to enzymatic degradation, lead to shorter half-lives. Thus, chemically modified to D-peptides have brought greater stability within GIT and systemic circulation.

### Addition of effective agents

#### Absorption enhancers

Absorption enhancers are usually one of a varied class of chemical moieties, they are used to improve drug absorption by facilitating intestinal cells permeation 37, 38. Generally, absorption enhancers alter the structural integrity of the epithelium or by simply promoting drug diffusion across the intestinal mucosa 39. The associated mechanisms of action which include: changing membrane fluidity or mucus viscosity, and/or opening tight junctions, generally governed by passive diffusion and modeled by Fick's first law of diffusion 39, 40.

The commonly used absorption enhancers are surfactants, fatty acids, chelators, glycerides, bile salts, salicylates, chitosan and cholesterol. They normally increase the solubility and bioadhesion of the drug or drug carrier system which allows more drug amount to be retained at the absorption site and resulting in greater drug oral bioavailability 41. However, it was found some absorption enhancers such as claudins, EDTA, sodium cholate, sodium dodecyl sulfate may cause the disruption of membrane integrity and systemic toxicity. The constant tight junction opening can cause mucosal damage and may also transport toxic molecules across the intestinal membrane 80. **Sadekar** *et al.* have developed an oral form of camptothecin by co-delivering with cationic, amine-terminated dendrimer, which is a promising intestinal mucosal penetration enhancer, drug solubilizers for oral drug delivery. The results showed camptothecin solubilization in gastric fluid and significantly enhanced oral drug absorption without opening the tight junction 38.

Sodium N-[8-(2-hydroxybenzoyl)amino]caprylate (SNAC) is a promising absorption enhancer can enhance passive permeation of polar charged drug molecules through the intestinal epithelium. This is noteworthy in view of the very low tendency of a polar drug to permeate over the lipophilic intestinal epithelial membrane 42. Several PPDs including calcitonin, insulin and heparin were conjugated with SNAC to promote the intestinal drug permeation 43. Semaglutide utilized this technique is in clinical trials, that has shown protection against gastric enzymes and enhanced hydrophobicity to promote the peptide drug permeate over the intestine. Additionally, SNAC has not been reported to be associated with significant disruption of the tight junctions, change in membrane fluidity, thus the low toxicity is beneficial for later clinical studies 42.

Another effective permeation enhancer, 8-(*N*-2-hydroxy-5-chloro-benzoyl)-amino-caprylic acid) (5-CNAC) is the leading examples of Eligen® technology from Emisphere. It was reported that 5-CNAC can deliver macromolecules (> 150 kDa), enhances transcellular absorption without disrupting intestinal integrity. **Karsdal** *et al.* incorporated 5-CNAC with calcitonin for oral administration. 5-CNAC interacts with calcitonin forming an insoluble entity at low pH in stomach, once it reaches small intestine at higher pH, the complex dissolves and facilitates intestinal drug uptake, resulting in systemic exposure of intact peptide 44. Currently there are ongoing trials for oral Eligen®- calcitonin for the treatment of osteoporosis 45. Moreover, Novo Nordisk's oral semaglutide which now has been marketed as tablet. Oral form of semaglutides, as glucagon-like peptide-1 (GLP-1) analogues, also utilises Emisphere Technologies' proprietary Eligen® Technology 46.

#### Modulation of pH

PPDs are usually formulated with enteric coating to prevent their degradation in the acidic environment. Once the enteric coating reaches the intestine, the increase in pH leads in dissolution of the coating and release the drugs, as was illustrated for an oral calcitonin form that has been tested in clinical trials 47. Moreover, Intestinal and pancreatic enzymes are also able to degrade PPDs in the neutral to basic environment in the small intestine. The use of citric acid in the oral PPDs formulation results in a decrease in pH, inhibiting degradation by the peptidases. **Lei** *et al.* have demonstrated that co-administration of citric acid reduced the activity of intestinal tryptic enzymes and resulted in higher oral bioavailability of calcitonin 48. However, the major concern is the distortion of physiological pH. Other limitations involve the long-term drug stability and the incompatibility upon dilution 49.

#### Proteolytic enzyme inhibitor

Direct inhibiting proteolytic enzyme by using an enzyme inhibitor is another way to circumvent intestinal enzyme activities. Proteolytic enzyme inhibitors such as aprotinin (inhibitor of trypsin and chymotrypsin), leupeptin (inhibitor for plasmin, trypsin, papain), chicken ovomucoid (trypsin inhibitor) and FK448 (chymotrypsin inhibitor). These proteolytic enzyme inhibitors are usually co-formulated with PPDs to prevent enzymatic degradation in intestinal mucosa. However, it was also reported that the safety of using enzyme inhibitors is a major concern. The excess use of this excipients may restrict certain therapeutic effects or trigger undesirable pharmacological activities 50, 51.

The most clinically advanced enzyme inhibition example is an oral insulin formulation known as ORMD-0801 consisting soybean trypsin inhibitor and a chelating agent that scavenges calcium. This treatment showed a significant 24.4% reduction in the frequencies of glucose readings >200 mg/dL, and a significant mean 16.6% decrease in glucose AUC 52.

#### Mucolytic agents

Mucolytic agents, also called mucus penetrating agents, which are able to facilitate the permeation of the drugs across the mucus barrier and elevate oral bioavailability of PPDs 53. In the reported preclinical studies, the use of PEG allows to promote mucus penetration 54. **Liu** *et al.* have developed a novel self-assembled nanoparticle composed of insulin and trimethyl chitosan, and a dissociable mucolytic agent. The mucolytic agent modified nanoparticles exhibited free Brownian motion and facilitate drug permeation over intestinal mucosa. In diabetic rats, the mucolytic agent modified nanoparticles generated a prominent hypoglycemic response and showed an bioavailability of 2.8-fold higher than that of unmodified nanoparticles. 55. While mucus-penetrating strategies continue to be extensively investigated, the efficacy and safety have not yet been validated in large clinical trials.

#### Cell-penetrating peptides

Cell-penetrating peptides (CPPs) are usually derived from viruses that are efficient at cell entry or membrane translocation, non-viral proteins or smaller molecules normally interact with membrane glycosaminoglycans, promoting PPDs to enter intestinal epithelial cells via endocytic pathways. However, the use of CPPs to elevate the oral bioavailability of PDDs has not yet been validated in the clinic 56.

Recently, CPPs such as HIV-1 Tat, penetration and oligoarginine are commonly used for oral delivery of various drugs 57, 58. **Kamei** *et al.* have used oligoarginine as a CPP to elevate the oral bioavailability of the peptide drug, leuprolide, the results found that leuprolide-oligoarginine conjugate attached to cell-surface proteoglycans and subsequently permeate over the ileal epithelial membrane via endocytosis pathway 59. However, inherent limitations were involved, including poor stability, toxicity and endosomal entrapment. To overcome this limitation, the enteric capsules can be used to avoid acidic and enzymatic degradation, thus promoting stability, and the sustain drug release of the CPPs modified formulation lower the toxicity of the CPPs to the intestinal mucosa 60.

### Drug carrier systems

#### Microparticulate carrier systems

Microparticles (size varying 1-100 µm) with high surface to volume ratio and greater intimate contact of the drugs with the intestinal epithelial layer, prolong gastric resident time, thus lead to higher drug absorption and oral bioavailability 61. For example, microparticles have shown that encapsulation of PPDs for oral administration and achieved a sustained biological activity. Surface modification of microparticles can be achieved by conjugation, coating or crosslinking. For example, collagen microparticles modified by photochemical crosslinking 62, and silk fibroin coated polylactide-co-glycolide acid (PLGA) and alginate microparticles have been used to further prolong the release of the peptide drug 63. **Onishi** *et al.* have developed enteric-coated chitosan-4-thio-butylamidine conjugate microparticles for oral delivery of calcitonin 64. **Yu** *et al.* have developed a glucose-responsive microsphere that could be used as an efficient insulin carrier for oral delivery, and resulted in sustained hypoglycemic effect 65. Several other new microparticulate systems have been developed recently. Such as temperature-responsive microspheres, dynamic hydrogel microspheres and glucose-responsive microspheres. However, the general limitations involve the polymer/drug miscibility, excipients compatibility for the system as well as the physical and chemical instability upon storage 66.

#### Hydrogels

Hydrogels generally contain water phase, a crosslinked polymer and a drug component. Usually they can respond to environmental changes to alter network structure, mechanical strength and swelling manner 67, 68. Generally, hydrogels remain insoluble even imbibe great amounts of biological fluids, therefore they appear to stabilize the embedded PPDs, protecting the PPDs from degradation in the harsh GI environment 69. In addition, the PPD loaded hydrogel is able to prolong retention time within specific gut regions thus elevate the drug absorption. However, hydrogels for oral delivery of PPDs have not made significant progress towards the clinical trials 68, 70.

**O'Neill** *et al.* have developed whey protein hydrogels encapsulating riboflavin. The dried microbeads hydrogel showed great resistance to GI degradation, underwent swelling and sustained release drug in GIT 71. Our team has previously developed a hydrogel using various mucoadhesive polymers to deliver glutathione. This polymeric hydrogel has shown great benefit for promoting the stability and bioavailability of the peptide drug 67. However, the main limitation of oral hydrogel is the physical and/or chemical instability issues, fast hydrogel disintegration may occur while it contacts with large amount of gut fluid after oral administration 69.

#### Nanoparticulate carrier systems

Nanoparticulate carrier systems, usually with particle size of less than 1 µm, such as polymeric or lipid nanoparticles, nanoemulsions and niosomes for oral drug delivery are of interest owing to the great benefit in promoting drug stability, provide a sustained drug release profile and elevate drug absorption over intestinal wall. In general, smaller particles of less than 500 nm are usually undergoes endocytosis and shows greater intestinal drug permeation than larger particles 72. During the process of endocytosis, the plasma membrane invaginates and pinches off to form enclosed vesicles and enter systemic circulation. Additionally, reducing the versicle size results in larger surface area, thus enhancing dissolution rate and solubility of PPDs However, limitations of nanoparticulate carrier systems are associated with limited drug loading and high particle aggregation due to thermodynamic instability, and scale-up difficulty for manufacturing 73.

**Fan** *et al.* have synthesized deoxycholic acid-conjugated chitosan, and loaded with the insulin into deoxycholic acid-modified nanoparticles (DNPs). It can overcome multiple intestinal barriers, internalized Caco-2 cells via apical sodium-dependent bile acid transporter (ASBT)-mediated endocytosis, and promoted the intracellular trafficking and basolateral release of insulin 74. **Lee** *et al.* developed a dual ligand functionalized pluronic-based nanoparticle for oral delivery of insulin. Chitosan and zonula occludins toxin (ZOT)-derived, tight junction opening peptide were conjugated to nanoparticles to increase the intestinal permeation of loaded insulin through the paracellular pathway 75. Our research team has also developed a PLGA based double emulsion nanoparticles for delivering glutathione. This nanoparticulate delivery system was able to elevate the drug retention time on mucosa, avoiding enzymatic degradation and promotes the transmucosal permeation of glutathione. However, the safety and biocompatibility of the polymeric materials and applicability of scaling up in manufacturing still remain a challenge 76.

#### Gold nanoparticle technology

Many publications have proposed the potential of gold nanoparticles (GNPs) for biomedical applications. The small size and multi-valence arrangement around the gold core elevates the capacity to improve drug biodistribution and hence effectiveness and safety 77. However, the GNPs that has entered clinical trials is CYT-6091 (Aurimune) is the only GNPs that have entered clinical trial currently. They are gold core particles incorporating TNF-α (a cytokine) and showed a particle size of 27 nm approximately. Studies demonstrated that incorporating TNF-α onto the gold platform improved systemic tolerability. In phase I studies, the safety profile showed the GNPs were well tolerated for patients with advanced cancer 78. Ultrasmall GNPs, with size of only 2-3 nm, have also showed great potential in a wide variety of therapeutic applications. It was demonstrated that ultrasmall GNPs with size around 2 nm have a relatively longer plasma half-life, improved tissue penetration compared with larger counterparts. Furthermore, ultrasmall GNPs offer a particularly high surface/volume ratio, which leads to greater dose-efficiency and all these indicated that it is a promising drug delivery vehicle for PPDs 79.

#### Microemulsion

Microemulsion is an isotropic, transparent and thermodynamically stable system which consists of water, oil and surfactant, usually with a co-surfactant. Droplet size is normally less than 200 nm. Structurally, they are divided into three phases: water-in-oil (W/O), oil-in-water (O/W) and bicontinuous microemulsion 80. Surfactants with a hydrophilic lipophilic balance (HLB) value greater than 12 are hydrophilic and predominantly forming O/W emulsions, while surfactants with HLB values less than 12 are favor in formation of W/O emulsion. Surfactants generally lower the surface tension to promote the drug solubility and opening tight junctions momentarily to enhance drug permeability. Moreover, surfactants having HLB greater than 20 usually require the addition of co-surfactants. However, some surfactants may cause some degree of toxicity, thus the amount of surfactant used requires careful consideration. Other limitations include the disintegration of the system due to dilution in the gut, and *in vivo* instability below the critical micelle concentration 81, 82.

**Momoh** *et al.* and **Erel** *et al.* have developed nano-encapsulated mucin and chitosan nanoparticles in W/O microemulsion for oral insulin delivery. They showed high entrapment efficiency and stability, sustained drug release and elevated intestinal permeation 83, 84. In recent years, self-microemulsifying drug delivery systems (SMEDDS), which emulsify spontaneously when exposed to the GIT fluid have been receiving increased attention. However, the low drug loading and the amounts of surfactant/co-surfactants used are limiting its application. Our group has previously developed a bicontinuous microemulsion for oral delivery of beta-carotene, which is a peptide drugs with very poor solubility. The optimized oral microemulsion promotes the stability and allows solubilizing beta-carotene, is a promising basis for further development as a functional beverage, as well as an oral delivery system for poor solubility peptide drugs 85.

#### Ionic liquid

Ionic liquids as low melting salts with melting point <100°C, often formulated to enhance the dissolution of poorly soluble drugs, as well as to promote drug permeation through physiological barriers. In general, ionic liquids interact with various hydrophilic and hydrophobic amino acids of a protein through an intricate balance of hydrogen bonds, disulfide bonds, ionic interactions and hydrophobic effects. When mix with water or body fluid, a more complex interplay between ions occurs, which can result in formation of microemulsions or micelles 86. **Williams** *et al.* developed an ionic liquid-based formulation for oral delivery of insulin, and the system showed high drug loading, a better access to the intestinal absorptive surface and prevented enzymatic degradation. Molecular dynamics simulation studies have shown that ionic liquids can remove water from the surface of enzymes to the same extent as polar organic solvents like acetonitrile. However, the safety issue is the major concern and the bulking care is essential particularly during handling and transport 87. **Banerjee** *et al.* have developed an ionic liquid-based oral formulation of insulin. This biocompatible delivery system has good long-term stability and facilitates intestinal absorption *via* paracellular uptake through the opening of tight junctions, results in promising insulin oral bioavailability. Thus, ionic liquids present an unprecedented and under-explored therapeutic opportunity with immense potentials for oral delivery of PPDs 87.

#### Liposomes

**Figure 4:**
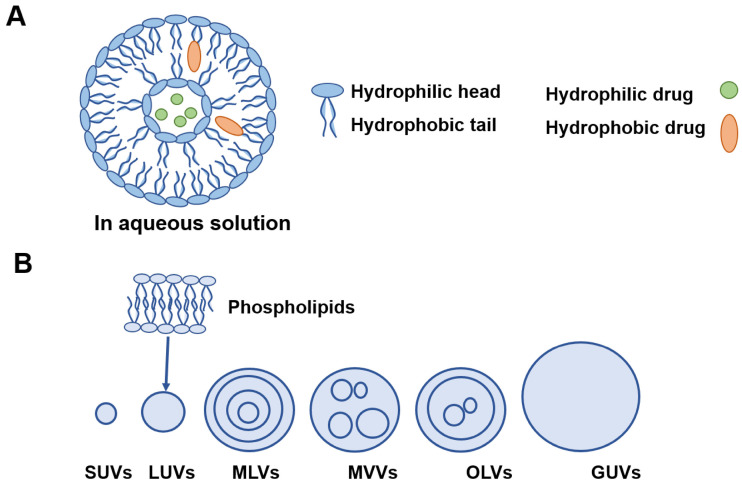
A) Basic liposome structure. B) Different model membranes of liposomes. SUVs: small unilamellar vesicles; LUVs: large unilamellar vesicles; MLVs: multilamellar vesicles; MVVs: multivesicular vesicles; OLVs: oligolamellar vesicles; GUVs: giant unilamellar vesicles.

Liposomes are generally composed of one or more phospholipid membrane bilayers surrounding aqueous inner phase with sizes from 15 nm to 10 µm 48 (**Figure 4A**).

Liposomes can be divided into six types based on their size and structures as shown in **Figure 4B**. Lipophilic drugs are embedded in the phospholipid layers while hydrophilic molecules are encapsulated in the aqueous inner core. This nature of liposomes that can carry both water soluble and lipid soluble drugs is called amphiphilic 88. **Suzuki** *et al.* have prepared a chondroitin sulfate-g-glycocholic acid-coated liposomes for oral exendin-4 (Ex-4) delivery. The long term pharmacodynamic effects, of daily oral exendin-4 loaded liposomes (300 μg/kg) were better than daily subcutaneous administration of Ex-4 solution (20 μg/kg) over 4 weeks 89. **Wang** *et al.* have used bovine serum albumin (BSA) adsorbed to cationic liposomes (CLs) to form protein corona liposomes (PcCLs) for oral delivery of insulin. The results showed great intestinal permeation, led to an increase of drug oral bioavailability and hypoglycemic effect 90. Our group has previously developed a deformable liposome to encapsulate catechin, which is a peptide drug extracted from green tea leaf, that can be easily undergo hydrolysis. The developed liposomes demonstrated great protection for the peptide drugs and elevated the bioavailability significantly 91. However, the major limitations involve poor stability, drug leakage of liposomes and short shelf life. The intact liposomes are difficult to permeate over the lipophilic intestinal epithelium, thus lower the oral bioavailability, especially for BCS class Ⅲ drug 92.

### Medical devices

#### Biodegradable microneedle-based delivery system

The inherent attractiveness of microneedle-based delivery strategy demonstrates the great suitability for various PPDs delivery, even with large molecular weight 93. **Prausnitz** *et al.* have utilized microneedle technology for oral drug delivery. They placed a 0.5-cm^2^ drug loaded microneedle patch onto the arms connected to a base, and called this device a luminal unfolding microneedle injector (LUMI). Once the oral administered device reached the intestine, the polymeric material holding the spring was dissolved, led to actuation that pushed the LUMI out, pressing the microneedle patches against the intestinal wall, allowing the drugs directly penetrate the intestinal epithelium. The Rani Therapeutics company has developed a related technology that deployed oral microneedles that has been carried out in a clinical trial currently, using octreotide as a model drug. Moreover, up to 0.3 mg of drug can be loaded into LUMI, which is sufficient for many potent PPDs 94.

Recently, it has been reported the preclinical studies of two oral microneedle devices, a poly(methacrylic acid-co-ethyl acrylate) and PEG based microneedle device for oral insulin delivery. The microneedle capsule was designed to dissolve at pH levels encountered in the small intestine. The results showed the insulin levels instantly increased and the blood glucose was reduced within 30 min, with an oral bioavailability of over 10% 95.

#### Ingestible self-orienting system

**Figure 5:**
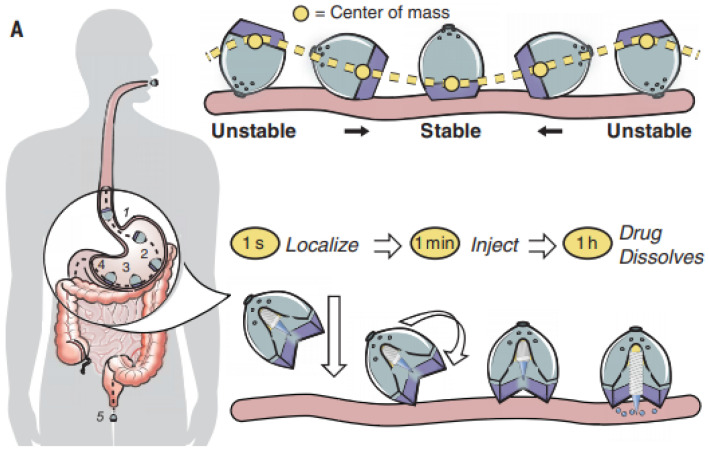
The ingestible self-orienting millimeter-scale applicator after oral administration, and the device could autonomously position itself to the intestinal mucosa. (Adapted with permission from 97, copyright 2021.)

An ingestible self-orienting system is a recent invented device that physically inserts a drug-loaded millipost through the GI mucosa with promising bioavailability. Inspired by the self-orienting leopard tortoise, **Abramson** *et al.* have developed an ingestible self-orienting millimeter-scale applicator (SOMA) that tends to position itself to engage with GIT, designed to resist external forces such as fluid flow, peristaltic motion upon reaching a stable point on the GIT wall. It then deploys milliposts fabricated from drugs directly through the intestinal mucosa while avoiding perforation. Figure 5 demonstrates the device positions to the stomach lining, orients its injection of the drug payload toward the GIT wall 96. This SOMA device has demonstrated promising efficacy to deliver insulin orally and could be used to deliver other PPDs orally. However, the drawback involves the deliverable dose is constrained by the formulation, volume and stability of the millipost. By increasing the size of millipost can elevate drug loading but might compromise the intestinal mucosa and trigger perforation risk. Furthermore, the long-term chronic effects brought by daily gastric injections shall be evaluated. Still, the SOMA represents a great platform for oral delivery of PPDs 97.

#### Intestinal mucoadhesive patches

Intestinal patches consist polymeric matrix embedding drugs, usually with a stabilizer. They can adhere to the intestinal wall and positioning the drugs directly to the intestinal epithelium, and meanwhile protecting the drugs from local enzymatic degradation 98. Recently, **Banerjee** *et al.* have fabricated an insulin loaded mucoadhesive oral patches integrated with iontophoretic circuit and surgically placed in the intestine. It was found the iontophoresis could disrupt the tight junctions of intestinal epithelium and facilitate insulin transport via paracellular pathway, without impairment of the intestinal mucosa. However, clinical proof of oral patch technology has not yet been forthcoming. However, the limiting drug loading and stability issue upon storage shall be considered 98, 99. Our research team has previously developed a mucoadhesive polymers‐based patch as a carrier system for delivery of glutathione. Various mucoadhesive biomimetic polymers were screen and the mucoadhesive patch was prepared using a simple casting method, and without using other unnecessary excipients. The optimal mucoadhesive patch has shown great potential for oral delivery of glutathione and other PPDs 100.

## Formulation technology with combinational strategies

**Figure 6:**
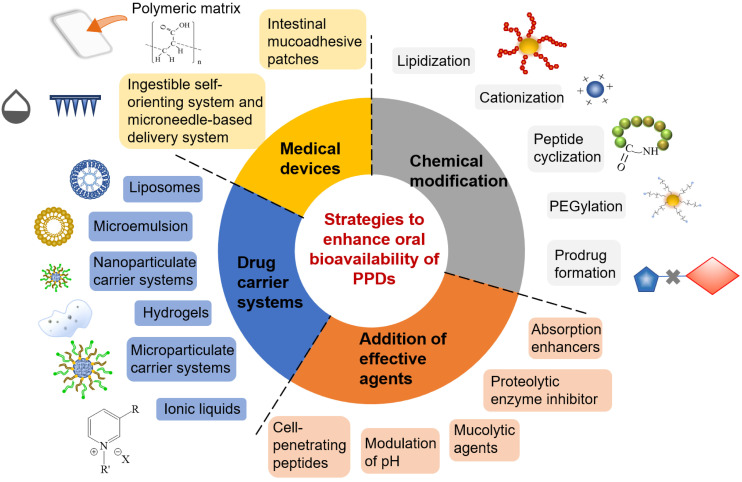
The overview of main formulation strategies for oral delivery of PPDs, including chemical modification, addition of effective agents, drug carrier systems and medical devices.

The following are some of the drug delivery technologies that utilize combinational strategies mentioned above (Figure 6), in order to advance and accelerate the oral absorption of PPDs. These are the successful examples with combinational strategies that are either in preclinical stage or at ongoing clinical settings are summarized below.

### Transient Permeation Enhancer^®^ (TPE^®^)

TPE^®^ had been used for oral delivery of octreotide. TPE^®^ is an oily suspension of octreotide that consists a permeation enhancer that can transiently modify the integrity of intestinal epithelium by opening the tight junction. It also consists polysorbate-80, allow to alter the thickness of intestinal mucus, thus further promote the intestinal drug uptake. Moreover, several peptides have been incorporated into TPE^®^ including teriparatide, leuprolide, insulin and octreotide. However, a main concern in application of TPE^®^, the intestinal tight junction opening that cause toxicity, or the use of food emulsifiers or other excipients might initiate autoimmune disease 101, 102. Currently, Phase I studies of octreotide capsules resulted in an oral bioavailability of about 0.7% and primary endpoints were achieved in two Phase III studies. The oral octreotide dose required to achieve these endpoints was over 200 times that of the 0.1 mg subcutaneous injection, which demonstrated a big achievement of this promising oral form 101.

### Gastrointestinal Permeation Enhancement Technology (GIPET^®^)

GIPET^®^ is an oral solid dose technology can effectively increase oral absorption of a variety of low permeability PPDs. This strategy focuses on the use of medium chain fatty acid or its variants coupled with salts, resulting in greater hydrophobicity and penetration characteristics that open epithelial tight junction 103. This technology is low cost and safe, which has great advanced to the clinic. GIPET^®^ consists three major enteric coated formats. GIPET^®^ I, is an enteric coated tablet with drug in selected weight ratios. GIPET^®^ II, is a microemulsion form encapsulated within an enteric coated gel capsule. GIPET^®^ III, consists of drugs with fatty acid derivatives within an enteric coated gel capsule. Currently, the Phase I and II studies have shown the safety profile of the three formats given on a repeated basis 104. In addition, permeation enhancer C_10_, have been incorporated to increase intestinal membrane fluidity and promote transcellular drug transport. Moreover, another feature of GIPET^®^ promotes the oral bioavailability of drugs may relate to inhibition of P-gp efflux 103.

### Peptelligence technology

Peptelligence^TM^ is a highly developed, clinically proven platform technology that enables the oral delivery of PPDs. It protects PPDs from acid hydrolysis, enzymatic degradation, and also enhances paracellular transport 105. Enteris's Peptelligence technology focuses on two main strategies, the first is a permeation enhancer, which opens tight junctions and facilitates paracellular transport. Second is a pH-lowering agent, lowering the local pH of the intestinal fluids in order to reduce protease activity. Additionally, the coating of the organic acid granules forms a thin barrier that prevents PPDs from acid degradation in the stomach 106. This technology was initially developed by Unigene and then Enteris Biopharma (Boonton, NJ, USA). Enteris has demonstrated positive results in several clinical studies, including phase III oral calcitonin and phase I oral leuprolide. The results from multiple preclinical as well as early and late-stage clinical studies have demonstrated the promising applicability of Peptelligence^TM^ to the oral delivery of PPDs 107.

### ThioMatrix™ technology

Thiolated mucoadhesive polymers (thiomers) that are capable of forming covalent bonds with intestinal mucus glycoproteins via thiol/disulfide exchange reactions. Thus, thiomers modified delivery system enhances the intestinal mucoadhesion, prolongs the retention in GIT and lead to higher oral bioavailability. In addition, thiomers also exhibit enzyme inhibitory, permeation enhancing and efflux pump inhibitory properties. However, thiomers are rather unstable in formulation form as they are subject of thiol oxidation at pH ≥ 5 unless sealed under inert conditions. Therefore, the use of pre-activated thiol groups might be an interesting approach to enhance its stability 108. ThioMatrix™ GmbH (Vienna, Austria) uses thiomers incorporates with reduced glutathione, to enhance oral delivery of hydrophilic macromolecules based on inhibition of protein tyrosine phosphatase by thiol groups. The results demonstrated the thiomeric mucoadhesive, permeation enhancing, and efflux pump inhibition properties were promising, thus lays a great platform for oral delivery of PPDs 103.

### Transferrin-based recombinant fusion protein technology

Transferrin (Tf) is an endogenous serum protein that transports iron to cells expressing the Transferrin receptor (TfR) through TfR-mediated endocytosis. Studies have applied Tf to prepare drug carrier system to deliver PPDs, genes and poor soluble drugs to the target tissues including intestinal epithelium and blood brain barriers that abundantly express Tf receptors 109. **Melanie** *et al.* generated and expressed functionally active colony-stimulating factor (G-CSF) as a recombinant fusion protein incorporated with Tf to evaluate the function of Tf as a carrier for oral delivery of G-CSF. The results demonstrated that the Tf moiety of the fusion protein not only promoted the drug permeation over the GI epithelium, but also protected the drug from enzymatic degradation 110. Therefore, it demonstrates that a Tf-based recombinant fusion protein technology is a promising approach for future development of orally active PPDs.

### Oral sCT (Ostora™) technology

Oral sCT (Ostora™) is built around coated citric acid vesicles in a Eudragit^®^-coated capsule, and currently has completed Phase III, indicating it is a clinically advanced oral peptide format. Briefly, it uses lauroyl carnitine chloride as the permeation enhancer to promote intestinal drug permeation, and citric acid as a pH lowering agent, lowering pH to reduce protease activity, as well as encapsulating within a Eudgradit^®^ capsule to prevent the drugs from acidic degradation in the stomach 111. There are other platforms with clinical trial data: TPE (Chiasma), POD™ (Oramed), Eligen^®^ (Emisphere), IN-105 (Biocon) and GIPET (Merrion). What stands out about these formulations is their simplicity compared with highly complex delivery constructs 111, 112.

### Oramed and Orasome technology

Oramed is a carrier system used for oral delivery insulin and GLP-1, which was developed by the Oramed Pharmaceuticals. Ormade's oral insulin is available as ORMD-0801, it allows to protect drug from enzymatic degradation and elevate the intestinal permeation of insulin. Ormades oral insulin was investigated for both type I and type II diabetes. It is currently under phase II clinical trial for oral insulin delivery and phase I trial for oral GLP-I delivery (NCT02535715) 113.

Orasome is a polymer-based liposome for oral delivery of insulin and human growth factor, which was introduced by the Endorex Corporation. This formulation allows to protect the loaded PPDs from acidic degradation in the stomach and protecting the drugs from the bile salts 114.

### Q-Sphera™ technology

Q-Sphera™ technology is a novel platform to individually print narrow size distribution particles of approximate 30 μm to generate predictable pharmacokinetic profile. This micro-piezo technology was developed by the MidaTech 115. Midatech's Q-Sphera technology focuses on long acting injectables using proprietary piezo printing technology that encapsulates PPDs into polymeric microparticles with precision properties. The piezo printing process regulates the internal pH inside microparticles and reduces the likelihood of protein destruction. Additionally, the Q-Sphere technique does not use surfactants, toxic solvents or biphasic mixtures, providing a promising safety profile of the technique. An example of Midatech's Q-Sphera has utilized an advanced 3D printing technology to fabricate a PLGA microparticle depot system. It is low cost and environmentally friendly, with an efficient high yield production and scalable manufacture 116.

### Nano Inclusion technology

This technology allows to solubilize potent molecules that have minimal solubility at biological pH for oral delivery 117. Midatech' MidaSolve project, MTX110, utilizes the MidaSolve nanosaccharide inclusion technology to solubilize panobinostat, allowing it to be orally administered via a micro-catheters system. Therefore, this technology focuses on promoting drug solubility, meanwhile the delivery system also elevates the oral drug bioavailability as well as to facilitate the drug to cross the blood-brain-barrier. The initial Phase I study showed promising safety profile in patients. Phase II trial of safety, tolerability, recommended dose and efficacy in 19 patients are under investigation. The study endpoint is expected to be patient survival after 12 months 118.

### Oleotec™ and Soctec™ gastro-retentive technology

Oleotec™ and Soctec™ gastro-retentive technologies were introduced by the Skyepharma. This strategy mainly focuses on promoting the drugs being absorbed in the stomach. Briefly, the technique prolongs the retention of the drugs within the stomach, and gradually releasing the encapsulated drug without being degraded by the acidic environment 119. Upon oral administrated the formulated dosage, the delivery system encapsulating drug was activated by GIT fluid. The polymer gradually swelled and enlarged 8 and 10 times in size, which guaranteeing its preservation in the stomach even after 6 - 8 hours of gastric emptying and released drugs in a sustained manner 120. The Accordion Pill™ is a typical gastro retentive formulation composed of polymeric films. It has a planar structure with multi-layer folded to an accordion shape, and encapsulated within a capsule. Upon reaching the stomach, the capsule dissolves, the Accordion Pill™ unfolds and allows to retain within the stomach for up to 12 hours 119.

## Targeting intestinal cell for oral PPDs delivery

**Figure 7:**
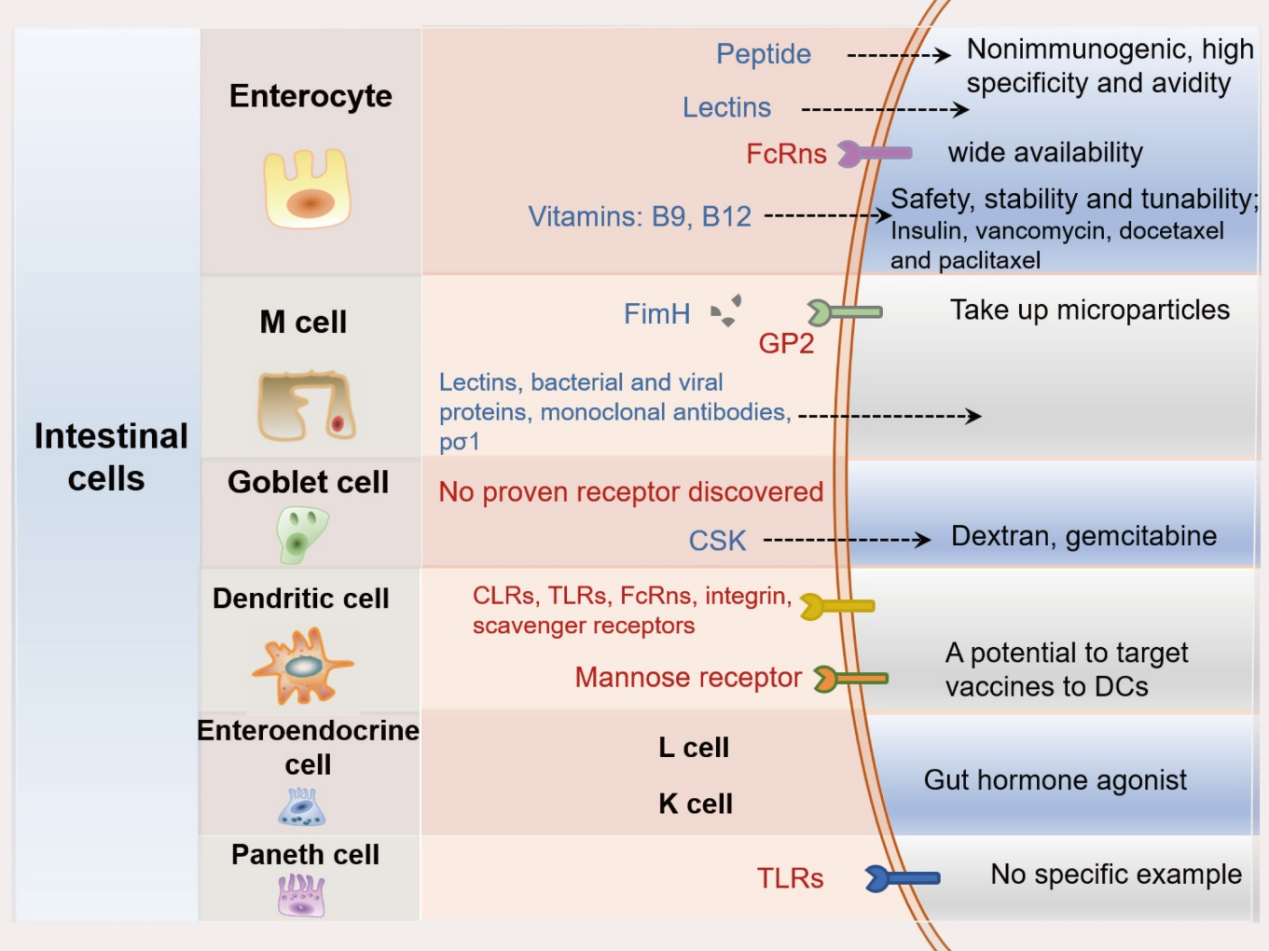
The overview of the intestinal cells-targeting strategies with the major cell types and the associated main receptors for oral delivery of PPDs.

A variety of intestinal cell types has been identified and characterized with different surface receptors which could be potential targets for oral PPDs delivery. The therapeutic applications of most PPDs largely depend on receptor-mediated endocytosis, and the relative affinity to these receptors are crucial. Therefore, targeting these stimulating endocytosis receptors on intestinal cell surface has drawn great attention for delivery of PPDs. For this purpose, surface modified drug delivery systems or ligand-grafted drugs are required. In the following sections, the use of ligands for targeting the major receptor of different types of intestinal cells will be discussed (Figure 7).

### Enterocyte targeting

Enterocytes are hyperpolarized epithelial cells with a columnar shape. They are the most prevalent cell type and are often targeted for the oral PPDs delivery. Several receptors have been reported to be expressing on the apical surface of enterocytes. Ligands, including vitamins, proteins, monoclonal antibody fragments and oligopeptides are often used for enterocyte targeting 129, 130.

Vitamins are commonly used ligands to decorate delivery systems for targeting specific intestinal cell receptors. Since they are very stable, safe with easy tunability. Vitamin B12 and biotin (vitamin B7) has been used for intestinal enterocyte targeting and showed promising results. Folic acid (vitamin B9) and thiamine have also been used as ligands for oral targeted delivery 131. Folic acid which enters enterocytes via a pH- and sodium ion-dependent pathway has been reported as efficient enterocyte-targeted ligands for the delivery of insulin and vancomycin 132. **Li** *et al.* used folic acid as a targeting ligand that grafted on nanoparticles to target the proton-couple folate transporter expressed on intestinal enterocytes, it mediated the endocytosis and facilitated the permeation over the intestinal mucosa 133. Owing to the abundant glycosylated proteins and lipids on intestinal enterocyte cells, lectins have great potential to promote cellular uptake of PPDs via specific binding 134. Peptides are particularly suitable as ligands because they are small, ease in synthesis and typically nonimmunogenic 135. **Zheng** *et al.* had developed an EGP peptide which targeted the heparan sulfate proteoglycans on the intestinal enterocytes. The EGP modified nanoparticles promoted the enterocytes uptake involving caveolae-mediated endocytosis and avoided lysosomal entrapment, thus facilitated the direct apical-to-basolateral transcytosis. Oral administrated insulin EGP NPs generated a strong hypoglycemic response on diabetic rats with 10.2-fold increase in bioavailability compared with free insulin 136. Further, the stability of peptides can be improved by simple modification, such as terminal blocking and insertion of D-amino acids 137. We have previously identified a PD-1/PD-L1 blocking D-peptide by using a liquid-phase phage display screening method, and it showed proteolysis-resistance and great stability *in vivo*, which is remarkably beneficial for its oral delivery 70. Arginine-glycine-aspartic acid (RGD) is widely used ligands to target integrin αvβ3 receptors, which are transmembrane glycoproteins overexpressed in intestinal Caco-2 cell line 138.

### Microfold cell targeting

Microfold cell (M cells) are one type of intestinal epithelial cells mainly located in the epithelium of Peyer's patches. Various types of cargo can be transported across M cells, such as antigens, bacteria, viruses and particles. Without passing through lysosomes, the cargo can avoid the lysosomal degradation which is a major benefit for transcytosis of the cargo. Furthermore, the undeveloped microvilli and glycocalyx structures of M cells allow the cargo to permeate over easier compared with the enterocytes 139. Owing to the above-mentioned features, M cell demonstrates as a promising target for oral PPDs delivery. The reported M cell targeting molecules include plant lectins, outer membrane bacterial proteins, and monoclonal antibodies. They were conjugated to the surface of delivery systems in order to improve the intestinal absorption of PPDs 140-143.

Glycoprotein 2 (GP2) is a highly expressed transcytotic receptor found on the intestinal M cells 144. FimH is an *Escherichia coli*-derived protein that showed GP2-targeting property. **Fan** *et al.* have developed a mucosal vaccine FimH-chitosan-pVP1 which exhibited great M cell-targeting capability, and this vaccine loaded FimH modified delivery system drastically uptakes by intestinal M cell, and promotes the dendritic cells maturation via TLR4-dependent signaling pathway 145. Lectin has been reported as another ligand to target M cells 146. Styrene maleic acid (SMA) nanomicelles were used to deliver epirubicin orally, which showed an increase of drug uptake without interrupting the intestinal membrane integrity. The SMA-micelles increased 2-fold drug accumulation in liver and spleen, and 6-fold and 15-fold higher accumulation in the lung and tumor, respectively. Additionally, SMA micelles showed colocalization with M cells and accumulation in Peyer's patches, which together confirms the M-cell mediated uptake and transport of SMA micelles 147. Moreover, ulex europaeus agglutinin-1 (UEA-1) is a fucose-specific lectin with an affinity for glycoproteins presented on M cells. UEA-1 modified carrier systems have demonstrated preferable uptake by M cells in mouse model, but their targeting ability in human M cells has not been clarified in clinical trials 147, 148. However, very limited number of ligands that could specifically bind to M cells were reported, especially to the transcytosis receptors.

### Goblet cell targeting

Goblet cells make up to 16% of the total intestinal epithelial cells and are responsible for producing mucins 149. So far, very few proven goblet cells targeting ligands were reported. **Jin** *et al.* have developed a trimethyl chitosan chloride (TMC) nanoparticle modified with CSK peptide for oral insulin delivery. The results showed the CSK peptide has significant effect on promoting drug permeation over intestinal epithelium, and the insulin loaded CSK modified nanoparticles produced a better hypoglycemic effect, with a 1.5-fold higher insulin oral bioavailability compared with drug solution 150. Previously, our research group had developed gemcitabine loaded CSK-TMC conjugates that significantly enhanced the drug uptake in mucus-producing cells due to the goblet cells targeting ability, and vastly elevated the oral drug bioavailability of 5.4-fold compared with plain drug solution 151. Moreover, a study demonstrated that wheat germ agglutinin (WGA) can bind to E-cadherin, which is also presented on mucus-secreting goblet cells. Interestingly, WGA modified nanocrystals were able to invade villi of goblet cells and reach lamina propria by transcytosis. The WGA modified nanocrystals showed an increased oral bioavailability of 17.5- and 2.4-folds higher than that of coarse crystals and plain drug, respectively 152. Therefore, the development of E-cadherin-targeting drug delivery systems also can be an alternative strategy for intestinal goblet cell targeting. Considering the large size and large number of goblet cells presented in the small intestine, thus it is worth investigating more specific targeting endocytosis-mediated receptors/transporters on goblet cells, as well as the more of the particular ligands targeting to them.

### Dendritic cell targeting

Dendritic cells (DCs) play a key role in protective immunity against pathogens 153. Intestinal DCs are a small subset of DCs that consist a large network in the intestinal immune system. DCs are distributed throughout the GIT, including the lamina propria of the intestine, mesenteric lymph nodes and Peyer's patches 154. It has been reported that mannose, Toll-like receptors (TLRs) and C-type lectin receptors (CLRs), integrins, neonata Fc receptors and scavenger receptors are the main endocytic receptors that expressed on the surface of DCs. Due to this variety of receptors, DCs have the ability to recognize various surrounding signals and induce immune responses 155, 156. Among the receptors, mannose receptors are the most popular receptor presented on the surface of DCs that offer the great potential for PPDs to target DCs. TLRs and CLRs were also proved as receptors to mediate cellular endocytosis. However, they also function as receptors to deliver activating signals to DCs via stimulating intracellular signaling cascades 157. Therefore, DCs targeting strategy is ideal for oral vaccine delivery. **Ramakrishna** *et al.* have successfully targeted the mannose receptor expressed on DCs using a fully human mannose receptor-specific antibody, B11, as a cargo to deliver human chorionic gonadotropin hormone. The results demonstrated B11 has great targeting capability toward DCs, and that mannose receptors and TLRs contribute towards activation and maturation of DCs by a mechanism that may be driven by a combination of peptide antigens and adjuvants 158.

### Enteroendocrine cell targeting

Enteroendocrine cells (EECs) are epithelial cells scattered throughout the whole GIT, which account for about 1% of the total intestinal cells 159. EECs constitute the largest endocrine system in our bodies, with over twenty different hormones that are secreted from intestinal EECs. Gut hormones physiologically regulate multiple biological effects, including intestinal motility and forming physical barrier for drug permeation. The apical membrane of enteroendocrine L and K cells expresses several receptors called G protein-coupled receptors (GPCRs), such as GPR40, GPR41, GPR43, GPR119 and GPR120. These receptors could be bound by dietary ligands such as carbohydrates, proteins, and lipids. These nutrients often stimulate the receptors and lead to secretion of enteroendocrine hormones 160, 161.

So far, very limited studies have focused in EEC targeting in oral drug delivery. **Nagatake** *et al.* reported that EECs expressed a tight junction membrane protein, claudin-4 (Cld4). Orally administered luminal antigens targeting Cld4 were found to be taken up by Cld4^+^ cells, indicating that Cld4-mediated transport can be a potential pathway for targeting delivery of PPDs. In addition, it was found that orally administered luminal antigens were taken up by the Cld4^+^ EECs, raising the possibility that EECs may also play a role in initiation of mucosal immunity 162. **Shrestha** *et al.* introduced a lipid-based nanoparticle which can act as endogenous ligands stimulating the release of GLP-1 via lipid-sensing pathways in enteroendocrine L cells 163. This study demonstrated that great potential of L cell targeting for treating GI disorders. **Xu** *et al.* have developed an innovative oral nanosystem to increase GLP-1 production and promote the oral absorption of peptides. The results showed the nanosystem triggered endogenous secretion of GLP-1 and increased its oral bioavailability by 4%. The nanosystem synergizes its own biological effect with the encapsulated peptide drug leading to a significant improvement of insulin resistance and glucose tolerance. This formulation strategy represents a promising approach for oral PPDs delivery in incretin-based diabetes treatment 121. Another study by Xu *et al*, the team has developed and compared different fatty acid-targeted nanocarriers and evaluated the L cell stimulation induced by the nanocarriers in vitro and in vivo. The results showed the DSPE-PEG_2000_ modified lipid-based nanocarriers had increased oral bioavailability of endogenous GLP-1 up to 8-fold in normoglycemic mice, and strengthened its biological effect 164.

### Paneth cell targeting

Paneth cells usually assist in maintaining the microbiome and are located at the crypts of intestinal villi. They have a longer survival time (up to 60 days) compared with enterocytes 165, suggesting their potential of being a good target for drug delivery. Toll-like receptor 9 (TLR9), is found to be expressed in Paneth cells, it recognizes bacterial DNA consisting unmethylated cytidine-phosphate-guanosine (CpG) dinucleotides. A study has reported that the oral delivery of oligonucleotides consisting a CpG sequence (CpG-ODNs) led to Paneth cell degranulation 166. **Rumio** *et al.* further studied the various TLRs presented on Paneth cells by orally delivering TLR agonists, and the results showed that the TLR3 agonist polyinosinic-polycytidylic acid also led to Paneth cell degranulation 166. However, these receptors are located in the endosome but not cell surface. Moreover, there is no published research that has exploit PDDs delivery by targeting Paneth cell and therefore the associated mechanisms underlying Paneth cell function are still unknown.

## Biomaterial with intestinal immune modulating function

The Peyer's patches of the small intestine play a central role in the intestinal immune system. The M cells of the Peyer's patches actively take up high-molecular weight components and are highly active in the process of phagocytosis and transcytosis. Thus, their main role is to take up luminal bacteria or large particles, transfer to DCs in the M-cell pocket for initiation of mucosal immune responses, and contribute to the homeostasis of the intestinal immune system 167. Moreover, as one of the main characters of M cells is that very limited lysosomes are presented within the cells, which lead to very low lysosomal enzyme degradation to the transporting cargo. Thus, the peptide antigens or other intact particles taken up by M cells are more easily transferred directly to DCs in the M-cell pockets or underneath the M-cells, and subsequently triggers immune-related activities, and modulating immune responses 168.

The new generation of polymeric biomaterials, which should be adaptive, complex, and intelligent. Thus, generally cover two main characteristics. First, biocompatible materials with controllable rigidity and functionality, forming polymeric biomaterials which can be widely used in oral drug delivery, tissue engineering, etc. Second, the biomaterials often “encoded” with information which could be read by proteins on intestinal cell surface, contributing significantly to intestinal cell-cell/cell-matrix communication. This information role of biomaterials can be demonstrated not only as intestinal cell targeting reagents but also as immune adjuvants in GIT 169.

As an intestinal immune adjuvant, most carbohydrates are widely found in plants, bacteria, and yeast. They are safe, low toxic, biodegradable, with high adjuvancy, thus, the carbohydrate-based adjuvants are promising candidates 170. Among all the carbohydrate-based materials, β-glucan is an excellent biomaterial to be used as drug carrier system with M-cell targeting property, as well as acting as an immunomodulating agent. β-glucan particles are spherical empty and highly purified cell walls of *Saccharomyces cerevisiae* and can be loaded with many classes of PPDs 171. In addition, β-glucans were predominantly observed in the Peyer's patches of the small intestine, suggesting that β-glucans are mainly taken up by intestine through M cells, which demonstrated its intestinal M cell-targeting property. Moreover, it was found that the oral administrated β-glucans particles were initially taken up by intestinal M cells, subsequently taken up by macrophages. These macrophages had expressed the β-glucan receptor Dectin-1, suggesting that it was taken up into the macrophages by phagocytosis through this receptor 172. These receptors recognize β-glucans particles and alert the host phagocytic immune cells and release of pro-inflammatory cytokines/chemokines. After maturation and migration of these antigen-presenting cells, T and B cell responses are also initiated 171, 173. Therefore, β-glucan is a biomaterial not just suitable to fabricate drug carrier system, also has intestinal M cell-targeting capability as well as immune modulating property.

Moreover, there are many other carbohydrate-based biomaterials, which have great intestinal bioadhesion, intestinal cell targeting properties, as well as intestinal immune modulating properties. There are many literatures reported that carbohydrates act as adjuvants through binding to specific innate immune receptors (e.g., TLRs, NOD2, C-type lectins, etc.), subsequently activate macrophages, DCs, NK cells, T lymphocytes or B lymphocytes, promoting the production of immune-related molecules, such as cytokines, antibodies, etc 169, 174. Recently, polysaccharides have caught scientists' attention, and many studies were employed polysaccharides as components of nanomaterials for modulation of the immune system. For example, mannan (α-MOS) can induce immune response by binding to CLRs (such as CD206) and TLRs. **Haddadi** *et al.* conjugated α-MOS with PLGA, the result showed the delivery system modified by α-MOS could promote phenotypic and functional maturation of DCs 169.

## Conclusions and future perspectives

The development of successful treatments involves not only the discovery of new therapies but also their adequate delivery to their targets. In this context, oral delivery of PPDs remains being a highly challenging endeavor. If we take into account that the first attempt to administer insulin orally was carried out in the 1920s and that, so far, there are very limited marketed oral formulations containing such large molecules, this enterprise may seem rather disappointing (Table 1). Various approaches have been developed for oral delivery of PPDs, including chemical modification on PPDs, co-administration with absorption enhancers and utilization of drug carriers or medical devices. Drug delivery systems targeted to various intestinal cell types are one of the most exploited strategies in the oral PPDs delivery. Although oral PPDs formulation approaches confer some significant advantages, more research is required given that the transition of these approaches from the bench to the bedside is associated with many challenges. This is partly caused by the physicochemical properties of PPDs with complex intrinsic nature, which can even lead to immunogenic reactions, and partly by GIT barriers, which related to enzyme secretion and physiology that are unavoidable.

Advanced delivery systems with novel biocompatible material and potential ligands, have demonstrated great potential in targeting different intestinal cells. However, limited numbers of receptors and ligands are available. In-depth understandings of the GIT biology in the molecular level are crucial for the discovery of new potential new receptor-ligand pairs. Based on the nature of disease and PPDs, single or combined receptor-ligand pairs could be used for intestinal cell targeting in future applications. Additionally, the cellular uptake pathways of oral drug delivery systems have not been comprehensively understood, and that poses gaps in knowledge regarding the interaction of PPDs delivery systems with the GI barriers and the dynamics PPDs metabolism. Further, as in most of the studies described herein, sustainable and tunable drug release for PPDs is still a challenge. The development of novel biocompatible materials with stimuli-responsive ability could be a potential solution. As a crucial type of biomaterial, we consider carbohydrates not only as matter or a structural component but also as information or signaling molecules. Although most of the discussed applications are still far from clinical use, carbohydrates deserve to be developed into next-generation biomaterials for oral drug delivery systems with great potential.

Lastly, even though multiple intestinal cells targeting delivery systems showed great potentials for oral delivery of PPDs, and several formulations are currently in advanced clinical trials, and disruptive novel technologies questioning previously established ideas have been proposed (Table 2). However, moving the applications from benchtop to bedside is still the biggest challenge, considering the cost and complexity of to accommodate the growing pool of PPDs. To help with the clinical transition of these approaches, standardization of preclinical parameters and procedures, integrative technology designs considering translational aspects, and knowledge sharing. Preclinical *in vitro* and *in vivo* studies could be performed under uniform conditions to enable accurate comparisons of various approaches. Thus, the future lies in tackling these hurdles and exploiting these novel approaches for oral PPDs delivery in the clinic.

## Figures and Tables

**Table 1:** Examples of formulation strategies of oral insulin with advantages and disadvantages.

Oral formulation techniques	Advantage	Disadvantage	References
Liposomes (e.g. HDV-1)	Superior mucus-penetrating capability; Excellent intestinal epithelial absorption.	Poor stability	89, 121, 122
Microemulsion	Improve the encapsulation efficiency.	Large particle size may exist	89, 121, 122
Nanoparticulate carrier system (e.g. Oshadi oral insulin)	High insulin loading; Promoted insulin intestinal permeation.	Complex preparation process; May lead to cytotoxicity	123
Hydrogels	Great stability, rapid response rate; High elasticity, and good biocompatibility.	Lack controlled release manner under different pH	89, 121, 122
Hydrogels and Cell-penetrating peptides	Controlled release manner; Permeation stimulatory effect	Stability issue within GIT	123
Microparticulate	High encapsulation efficiency	Large particle size leads to poor absorption	89, 121, 122
Absorption enhancers (e.g. ORMD-0801, IN-105, Oshadi oral insulin)	Protecting against enzymatic degradation; Improving drug absorption	Risk of infections.	124-126
pH sensitive enteric coating (e.g. ORMD-0801, Capsulin)	Protect the drug from pepsin hydrolysis; Sustained released and greater drug absorption.	Difficulties in oral administration for infants or younger children.	124, 127
Insulin modification (e.g. IN-105)	Protect drug from enzyme and acid degradation; Controlled release manner	Identify suitable modification sites. Bioactivity may be reduced after modification.	126, 128

**Table 2:** Current clinical status of major PPDs for oral administration.

Protein/Peptide	Conditions or diseases	Delivery approach	ClinicalTrials.gov identifier
Homeopathic antibodies to the TLR3 FYW peptide (TAO1)	●Common Cold	Impregnation of pre-made tablets	NCT01651715 (Phase I/ Phase II)
Anti-CD3 monoclonal antibody	●Chronic Hepatitis C	Neutralize stomach pH for enhancing stability of the Mab with Omeprazole	NCT01459419 (Phase II)
	● Nonalcoholic Steatohepatitis		NCT01205087 (Phase II)
Insulin	●Diabetes Mellitus, Type 1	pH sensitive Capsules	NCT02580877 (Phase II); NCT00419562 (Phase III); NCT02535715 (Phase II);
	●Diabetes Mellitus, Type 2	pH sensitive Capsules and enzyme inhibition	NCT02954601 (Phase II); NCT01889667 (Phase II);
	●Brittle Type I Diabetes Mellitus	pH sensitive Capsules and enzyme inhibition	NCT00867594 (Phase II)
	●Nonalcoholic Steatohepatitis	pH sensitive Capsules and enzyme inhibition	NCT04616014 (Phase II);
	●Diabetes	Hepatic directed vesicles	NCT00814294 ((Phase II/Phase III)); NCT00521378
	●Diabetes Mellitus, Type 1	Insulin modification and enhanced osmosis	NCT01035801 (Phase I)
	●Diabetes Mellitus, Type 2	Insulin modification and enhanced osmosis	NCT03392961 (Phase I); NCT03430856 ((Phase II/Phase III)
	●Insulin-Dependent ●Diabetes Mellitus	Nanoparticle encapsulation and permeability enhancement	NCT01120912 (Phase I); NCT01973920 (Phase II); NCT01772251 (Phase I/ Phase II)
Glucagon like peptide-1 Analogue	●Diabetes	Permeation enhancer	NCT02094521 (Phase I)
Leuprolide	●Endometriosis	Permeation enhancer, pH modulator and enzyme inhibitor	NCT05096065 (Phase II)
Salmon calcitonin	●Osteopenia	Antiproteolysis and absorption enhancement	NCT01292187 (Phase II); NCT00959764 (Phase III)
Acyline	●Contraception	Gastrointestinal permeation enhancement	NCT00603187 (Phase I/ Phase II)
Dolcanatide	●Constipation	Chemical modification	NCT01983306 (Phase II)
Parathyroid hormone (1-34)	●Hypoparathyroidism	Permeation enhancers and enzyme inhibitor	NCT02152228 (Phase II)
Cyclosporine A (CSA)	●Ulcerative Colitis	Emulsion (Oil-in-water)	NCT01033305 (Phase II)
